# Synaptic determinants of cholinergic interneurons hyperactivity during parkinsonism

**DOI:** 10.3389/fnsyn.2022.945816

**Published:** 2022-09-06

**Authors:** Montserrat Padilla-Orozco, Mariana Duhne, Alejandra Fuentes-Serrano, Aidán Ortega, Elvira Galarraga, José Bargas, Esther Lara-González

**Affiliations:** ^1^División Neurociencias, Instituto de Fisiología Celular, Universidad Nacional Autónoma de México, Mexico City, Mexico; ^2^Department of Neurology, University of California, San Francisco, San Francisco, CA, United States; ^3^Department of Neuroscience, Feinberg School of Medicine, Northwestern University, Chicago, IL, United States

**Keywords:** Parkinson’s disease, cholinergic interneurons, striatal microcircuit, electrophysiology, calcium imaging

## Abstract

Parkinson’s disease is a neurodegenerative ailment generated by the loss of dopamine in the basal ganglia, mainly in the striatum. The disease courses with increased striatal levels of acetylcholine, disrupting the balance among these modulatory transmitters. These modifications disturb the excitatory and inhibitory balance in the striatal circuitry, as reflected in the activity of projection striatal neurons. In addition, changes in the firing pattern of striatal tonically active interneurons during the disease, including cholinergic interneurons (CINs), are being searched. Dopamine-depleted striatal circuits exhibit pathological hyperactivity as compared to controls. One aim of this study was to show how striatal CINs contribute to this hyperactivity. A second aim was to show the contribution of extrinsic synaptic inputs to striatal CINs hyperactivity. Electrophysiological and calcium imaging recordings in Cre-mice allowed us to evaluate the activity of dozens of identified CINs with single-cell resolution in *ex vivo* brain slices. CINs show hyperactivity with bursts and silences in the dopamine-depleted striatum. We confirmed that the intrinsic differences between the activity of control and dopamine-depleted CINs are one source of their hyperactivity. We also show that a great part of this hyperactivity and firing pattern change is a product of extrinsic synaptic inputs, targeting CINs. Both glutamatergic and GABAergic inputs are essential to sustain hyperactivity. In addition, cholinergic transmission through nicotinic receptors also participates, suggesting that the joint activity of CINs drives the phenomenon; since striatal CINs express nicotinic receptors, not expressed in striatal projection neurons. Therefore, CINs hyperactivity is the result of changes in intrinsic properties and excitatory and inhibitory inputs, in addition to the modification of local circuitry due to cholinergic nicotinic transmission. We conclude that CINs are the main drivers of the pathological hyperactivity present in the striatum that is depleted of dopamine, and this is, in part, a result of extrinsic synaptic inputs. These results show that CINs may be a main therapeutic target to treat Parkinson’s disease by intervening in their synaptic inputs.

## Introduction

A decrease in dopamine in the basal ganglia, the cortex, and the thalamus, due to the death of dopaminergic neurons, is a hallmark of Parkinson’s disease (Dauer and Przedborski, 2003; Maiti et al., 2017). As a result, the striatum exhibits a significant and generalized increase in spontaneous activity and neural synchrony during Parkinsonism (Carrillo-Reid et al., 2008; Jáidar et al., 2010, 2019; Plata et al., 2013a; Pérez-Ortega et al., 2016; Aparicio-Juárez et al., 2019; Lara-González et al., 2019). This altered activity underlies the origin of abnormal oscillations linked to motor deficits (Brown, 2007; Little and Brown, 2014; Feingold et al., 2015; Deffains and Bergman, 2019) and striatal tonically active neurons (TANs), mainly cholinergic interneurons (CINs), are involved in this process (Raz et al., 1996, 2001; Kondabolu et al., 2016). There are two classes of striatal TANs: low-threshold spiking interneurons (LTSIs) and CINs. Focus on CINs is supported by the fact that dopamine (DA) depletion is accompanied by hypercholinergy, a cardinal feature of Parkinsonism, thought to be a consequence of CINs’ altered activity (Barbeau, 1962; Galarraga et al., 1999; Tanimura et al., 2017; Abudukeyoumu et al., 2018; Ztaou and Amalric, 2019). In addition, optogenetic inhibition of CINs results in a temporary suppression of oscillations (Kondabolu et al., 2016) and motor signs of the disorder (Maurice et al., 2015). Notwithstanding, some investigators reported that CINs activity decreases during dopamine (DA) depletion (McKinley et al., 2019; Choi et al., 2020), while others reported that CINs activity increases in this condition (Sanchez et al., 2011; Tubert et al., 2016; Tubert and Murer, 2020; Paz et al., 2021).

CINs are crucial in the regulation of excitatory and inhibitory balance across the striatum (Galarraga et al., 1999; Carrillo-Reid et al., 2009). They anatomically constitute 1–2% of striatal neurons and are the main source of acetylcholine in this nucleus (McGeer et al., 1971; Butcher and Butcher, 1974; Phelps et al., 1985; Wilson et al., 1990; Zhou et al., 2002; Dautan et al., 2014). They are distinguished from the rest of the striatal neurons by the large diameter of their somata, which can exceed 40 μm (Lapper and Bolam, 1992; Gerfen and Bolam, 2016), and their unique electrophysiological characteristics that include regular or irregular single spike autonomous firing at a frequency of 2–10 Hz and bursting interspersed with pauses (Wilson et al., 1990; Aosaki et al., 1995; Bennett and Wilson, 1999; Bennett et al., 2000; Goldberg and Wilson, 2005; Wilson, 2005; Tan and Bullock, 2008; Sanchez et al., 2011; Schulz and Reynolds, 2013). Autonomous firing involves persistent Na^+^, Ca^2+^, BK, and SK ion channels producing middle and longer duration after hyperpolarizations (mAHP, sAHP; Bennett et al., 2000; Goldberg and Wilson, 2005; Wilson and Goldberg, 2006), a prominent hyperpolarization-activated cation (HCN) current resulting in a “sag” and a rebound firing after hyperpolarization (Deng et al., 2007), resting membrane potential of about –60 mV (Bonsi et al., 2011; Sanchez et al., 2011), input resistance above 200 MΩ, and a long action potential duration of about 5 ms measured at mid-amplitude (Wilson et al., 1990; Fino et al., 2007; Tubert et al., 2016; Arias-García et al., 2017). Accordingly, CIN’s altered firing during Parkinsonism has been attributed to changes in CINs’ intrinsic properties, such as autoreceptor (M4) maladjustment (Ding et al., 2006), or HCN (Deng et al., 2007), and K^+^ channels dysfunctions (Sanchez et al., 2011; Tubert et al., 2016; Tubert and Murer, 2020). Here, we asked which of these changes can be observed while comparing control and DA-depleted CINs at similar membrane potential and firing frequencies.

Nevertheless, changes in the actions of excitatory and inhibitory synaptic inputs onto CINs during the Parkinsonian state have not been fully addressed, despite the important alterations in these striatal inputs (Galarraga et al., 1987; Calabresi et al., 1996; Henderson et al., 2000; Zhang et al., 2013; Smith et al., 2014; Villalba et al., 2015; Glajch et al., 2016; Parker et al., 2016; Aceves-Buendia et al., 2017; Zhai et al., 2018). Could it be that CINs are the main target for these inputs? In this study, we used multi-neuron recordings using calcium imaging plus electrophysiology in trying to understand the role of synaptic inputs on the firing of CINs during Parkinsonism. We used pharmacological antagonists of glutamatergic, GABAergic, and nicotinic inputs to explore whether there is a synaptic restructuration contributing to CINs pathological activity in the DA-depleted striatum. Our findings support the role of altered synaptic inputs to explain altered CINs firing.

## Materials and methods

### Animal use and care

Experimental subjects were B6; 129S6-Chat^TM2(*cre*) *Lowl*^/J (ChAT-cre mice; JAX stock: 006410) mice that were acquired from Jackson Laboratories,^1^ and mating was carried out between homozygous mice. A total of 144 male and female animals were used for control and DA-depleted experimental groups. Protocols were designed and performed as approved by the Institutional Committee for Laboratory Animals Care and Use of the Instituto de Fisiología Celular (IFC), UNAM (NOM-062-Z00-1999; laboratory protocols JBD-59-15) in accordance with the international norms for the ethical use of experimental animals established in the National Institutes of Health Guide for Care and Use of Laboratory Animals Eighth Edition (National Research Council, 2011). Mice were bred and housed in our animal facilities in a pathogen-free, temperature-controlled room, on a 12:12-h light–dark cycle, with food and water intake *ad libitum*. Experimental samples were drawn from animals expressing Syn-GCaMP6f and tdTomato-FLEX for calcium imaging and immunohistochemistry assays (*n* = 18 animals), GCaMP6f-FLEX to follow only ChAT+ neurons for calcium imaging experiments (*n* = 61 animals), or tdTomato-FLEX for electrophysiological recordings (*n* = 65 animals). Mice from each litter were randomly chosen for different samples.

### Dopamine depletion using neurotoxin 6-hydroxydopamine

The neurotoxin 6-hydroxydopamine (6-OHDA) has been used for a long time to obtain murine models of Parkinson’s disease. Its ability to produce massive destruction of nigrostriatal dopaminergic neurons by its infusion into the SNc has been used to investigate motor and biochemical dysfunctions of the disorder (Garcia-Munoz et al., 1983; Simola et al., 2007). For this study, 69 mice were anesthetized by intraperitoneal administration of ketamine (85 mg/kg)-xylazine (15 mg/kg) and prepared for stereotaxic surgery, in which 0.6 μL of 6-OHDA were injected at a rate of 0.2 μL/min. The coordinates used were –2.6 mm AP, –1.5 mm LM, and –4.7 mm DV from Bregma, with the SNc as the target. At the end of the process, the mice were placed on heating racks until they woke up. The health status of the mice was monitored in the following 15 days, during which postoperative care was administered, including the administration of antibiotics, analgesics, and anti-inflammatory drugs. When a weight loss greater than 20% was detected, the intervention was performed with a daily injection of 100 μL of 2% glucose in saline solution. After this period, the degree of DA depletion was assessed by turning behavior evaluation. For this purpose, apomorphine was injected subcutaneously (0.5 mg/kg in the saline vehicle with 0.02% ascorbate) and, subsequently, ipsi and contralateral turns to the lesion site were quantified using the RotaCount 2.0 software (Omni-Tech, Sioux Falls SD) for at least 60 min. Mice were considered a successful DA-depleted model when they exhibited more contralateral than ipsilateral rotations (at least 60 contralateral rotations per hour and few or no ipsilateral rotations). Lesion efficacy was 65% as measured by this test (see Lara-González et al., 2019). The injured hemispheres from 6-OHDA mice were used in some of the experiments described. Experiments were performed in well-lesioned mice 3–4 weeks after the turning behavior evaluation (Pollack et al., 1997). Note that no dopaminergic agonist was used in these experiments after the sole injection of apomorphine. Mice that failed the turning behavioral test because of a misplaced lesion and those with no lesions were used as controls. No differences were found between these two groups and their results were pooled together.

### Viral infection

Adeno-associated virus (AAV) vectors were used to either express GCaMP6f into ChAT+ neurons or co-express tdTomato into ChAT+ neurons plus GCaMP6f under synapsin promoter for expression in all neurons. Co-expression dilutions were always 1:1. Viral vector pAAV.Syn.GCaMP6f.WPRE.SV40 and pAAV.Syn.Flex.GCaMP6f.WPRE.SV40 were obtained from the Genetically Encoded Neuronal Indicator and Effector Project (GENIE) & Douglas Kim (Addgene viral prep #100837-AAV1^2^; RRID: Addgene_100837; Addgene viral prep # 100833-AAV1^3^; RRID: Addgene_100833, Dana et al., 2016). Viral vector AAV pCAG-FLEX-tdTomato-WPRE was a gift from Hongkui Zeng (Addgene viral prep # 51503-AAV1^4^; RRID: Addgene_51503). Mice were anesthetized by intraperitoneal injection of ketamine (85 mg/kg)-xylazine (15 mg/kg) solution to subsequently perform stereotaxic surgery by injecting 0.6 μL of viral vector (100 μL at titer ≥ 1 × 10^13^ vg/ml) at coordinates + 0.9 mm AP, –1.7 mm LM, and –2.8 mm DV from Bregma at a rate of 0.1 μL/min with a dental needle (corresponding to dorsolateral striatum). In this process, artificial tears were placed in the eyes of the mice to prevent damage. Body temperature was monitored perioperatively and postoperatively until the complete recovery of the animal. Mice recovered within 3 weeks, during which care actions were implemented to minimize pain and discomfort (see above). All *in vitro* experiments were performed in 75 control mice and 69 hemi-Parkinsonian mice.

### Slice preparation

After being anesthetized by intraperitoneal administration of ketamine (85 mg/kg)-xylazine (15 mg/kg), mice were perfused intracardially with cold sucrose solution (in mM): 234 sucrose, 28 NaHCO_3_, 7 dextrose, 4.54 pyruvate, 0.28 ascorbic acid, 2.5 KCl, 7 MgCl_2_, 1.44 NaH_2_PO_4_, and 0.4 CaCl_2_ at 4°C. pH = 7.4). The brains were extracted and hemispheres separated. A parahorizontal cut was made to the right hemisphere at an angle of 30°, after which 250-μm-thick slices were obtained using a vibratome (PELCO easiSlicer; Ted Pella, Redding, CA). Slices were kept in artificial cerebrospinal fluid (ACSF, with a composition in mM: 126 NaCl, 15 dextrose, 26 NaHCO_3_, 0.2 thiourea, 0.2 ascorbic acid, 2.5 KCl, 1.3 MgCl_2_, 1.2 NaH_2_PO_4_, 2.0 CaCl_2_, pH = 7.4; 300 ± 5 mOsm/L), perfused with 95% O_2_ and 5% CO_2_ at room temperature. Slices were placed under a 20X immersion objective (Olympus XLUMPLFLN Objective, 1 NA, 2 mm WD; New York) while constantly perfused with ACSF, 95% O_2_ and 5% CO_2_ for calcium imaging recordings. Both calcium imaging and electrophysiological recordings were performed at room temperature (25–30^°^C).

### Calcium imaging recordings

Fluorophore stimulation was carried out with a Lambda HPX High power LED driver coupled to specific excitation emission filters according to GCaMP6f parameters: excitation: 460–480 nm, emission: 495–540 nm (Olympus, U-MGFPHQ), and those corresponding to tdTomato: excitation 565/20 nm (Chroma Technology Corporation, D565/20 m), emission 610/75 nm (Chroma Technology Corporation, HQ610/75 m). Imaging recordings were obtained with a CoolSnap K4 camera, controlled by Im-Patch^©^, an open access software.^5^ ChAT+ neuron activity was observed in ChAT-cre mice using the GCaMP6f signal. A solution of KCl (15 mM) was administered at the end of all the experiments to determine the viability of the slices, analyzing only those that presented more than 85% of viable cells.

### Electrophysiological recordings

Besides identifying CINs from ChAT-cre mice with viral transfections, their activity was recorded to identify their electrophysiological profile, as previously described, in the absence of any neurotransmitter or channel antagonists (Aosaki et al., 1994; Bennett and Wilson, 1998, 1999; Bennett et al., 2000; Sanchez et al., 2011; Tubert et al., 2016), to compare an array of intrinsic properties, such as spontaneous firing rate, input resistance, and other parameters under control conditions and after dopamine (DA) depletion. We recorded inferred electrical activity with calcium imaging (6–10 frames per second) to verify the calcium indicator GCaMP6f efficiency to follow, as much as possible, the firing of neurons during different types of firing patterns. Inferred electrical activity was done as in previous studies (Carrillo-Reid et al., 2008; Jáidar et al., 2010, 2019; Pérez-Ortega et al., 2016), using the time derivative of the calcium transients. In both cases, whole-cell current-clamp configuration was carried out using patch pipettes (3–6 MΩ) filled with a solution containing (in mM) 115 KH_2_PO_4_, 2 MgCl_2_, 10 HEPES, 10 EGTA, 10 NaCl, 0.2 Mg^2 +^-ATP, and 0.2 Na-GTP, with pH = 7.24 and 290 ± 5 mOsm/L.

### Drugs used

The following are the drugs used: 6-cyano-2, 3-dihydroxy-7-nitro-quinoxaline disodium salt (CNQX, Sigma-Aldrich C239-25MG), D-(_)-2-amino-5-phosphonovaleric acid (APV, Sigma-Aldrich A8054-5MG), and 2-(3-carboxypropyl)-3-amino-6-(4 methoxyphenyl) pyridazinium bromide (Gabazine, Sigma-Aldrich S106-10MG) were used to block glutamatergic and GABAergic synaptic transmission. Mecamylamine hydrochloride (Sigma-Aldrich M9020-5MG) was used to block nicotinic receptors. Stock solutions were prepared in deionized water and added to the perfusion system during the experiment to obtain the required final concentration (Gabazine, CNQX, and Mecamylamine = 10 μM; APV = 50 μM). APV was prepared with dimethylsulfoxide (0.01%).

### Immunohistochemistry

After electrophysiological recordings, slices were fixed with the following: Phosphate-buffer saline (PBS) 4% PFA, 1% picric acid, and pH = 7.4 and stored for immunohistochemistry. Slices were incubated with BSA 1% in PBS (pH = 7.2) for 30 min. They were washed with PBS for 10 min for 3 times. The primary antibody was diluted in 1:1,000 in PBS, triton 2.5%, and applied for 24 h. Subsequently, slices were washed again for 10 min for three times in PBS. A secondary antibody (1:500 in PBS/triton) was applied for 1.5 h and later washed in PBS for 10 min three times. Slices were finally cover-slipped with a DAPI-mounting medium (Vectashield, Vector Laboratories, Inc., H-1200). Confocal images were acquired in a Zeiss LSM-710 (objective: C-Apochromat 20X N.A.45, GCaMP6f: 488 nm laser excitation, 493–533 nm emission; tdTomato: 543 nm laser excitation, 565–615 nm emission; Alexa 647–633 nm laser excitation, 658–704 nm emission). Antibodies were purchased as follows: ChAT (Millipore Cat# AB144P, RRID: AB_2079751)^6^ and Antigoat Alexa Fluor 647 (Jackson ImmunoResearch Labs Cat# 705-605-003, RRID: AB_2340436) from Jackson Mice, Maine, US).^7^

### Data analyses

Electrophysiological and calcium imaging recordings were processed using Im-Patch^©^ software and MATLAB^©^ (RRID: SCR_001622).^8^ For electrophysiological recordings, digitized data were imported for analysis and graphing into commercial software: Origin 7 (Microcal, Northampton, MA, United States); MatLab (Natick, MA, United States); Graphpad Prism 5 (San Jose, CA, United States) and Systat 11 (San Jose, CA, United States).

Electrophysiological data analyses: Intrinsic membrane properties allow us to compare different neuron samples. To measure whole cell input resistance (R_*N*_), we used current-voltage relationships (I-V plots). Since I-V plots were non-linear, we fitted a third-order polynomial to each of them. Rheobase currents were measured as the minimum current necessary to generate one action potential (AP) in 1 s. The sag ratio was calculated by dividing the amplitude of the voltage change in response to a 1-s hyperpolarizing current pulse (–100 pA) at the beginning of the pulse divided by its value at the end of the pulse. Another action potential (AP) parameters were measured from spontaneous spikes at similar membrane potentials and frequencies in both control and Parkinsonian CINs so that electrical stimulation does not introduce additional bias by the activation/inactivation of different ionic conductance due to DA depletion (Ding et al., 2006; Deng et al., 2007; Tubert et al., 2016). However, intensity-frequency plots (I-F plots) in control and DA-depleted conditions illustrated in Supplementary Figure 1 support previously described changes in intrinsic properties (compared with two-way ANOVA with *post hoc* Tukey tests for multiple comparisons). AP properties, such as speed of depolarization and repolarization, AP-amplitude, and afterhyperpolarization (AHP) amplitude, were measured with dV/dt as a function of V plots [dV/dt(V)-plots]. AHP’s half-width duration was calculated at half of its amplitude during spontaneous firing at a similar voltage range (3–4 Hz) in both control and DA-depleted CINs. The number of APs during the response to a 1 s, 40 pA, depolarizing current steps was calculated (Mean frequency). Values are reported as mean ± SEM. The mean spontaneous firing rate and coefficient of variation of interspike intervals were determined by averaging 2 min recordings in control and DA-depleted CINs at similar membrane potential, as well as the frequency to check whether under these constraints, differences in intrinsic parameters still play a role.

**FIGURE 1:**
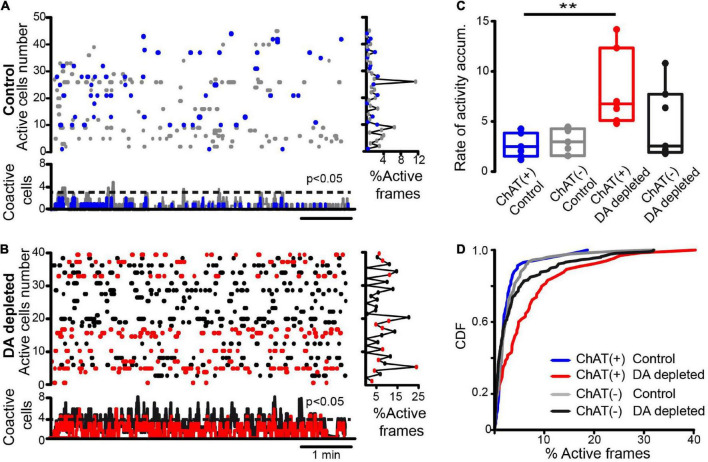
Spontaneous neuronal activity within striatal microcircuits in control and after dopamine depletion. **(A)** The raster plot shows the spontaneous activity of several neurons recorded simultaneously with calcium imaging in control conditions. Blue dots are identified CINs (tdTomato in ChAT-cre mice), and gray dots are presumably other neuron types. The right histogram shows activity per neuron (rows) and the histogram at the bottom shows coactivity (neurons that fire together in a column vector or movie frame). A dashed line indicates when a column vector is significant using Monte Carlo simulations (*P* < 0.05). In control, significant peaks of coactivity are scarce and rarely coincide with CINs peaks. **(B)** Similar raster plots showing the spontaneous activity of several neurons recorded simultaneously in DA-depleted striatum: notice increased activity as compared to the control. Red dots are identified CINs. The right histogram shows increased activity per row. Histogram of coactivity at bottom exhibits numerous significant peaks of coactivity along time, these are often accompanied with CINs activity. **(C)** Box plots show that the rate of activity accumulation of CINs over time increases after DA depletion (see Material and methods; Supplementary Figure 2; Kruskal-Wallis ANOVA with *post hoc* Dunn’s test: ChAT+ active neurons before (control) and after DA-depletion: **P = 0.0087). **(D)** Cumulative distribution functions (CDFs) of activity of ChAT+ and ChAT- neurons before and after DA depletion (control ChAT+ vs. control ChAT- neurons: *P* = 0.76; control ChAT+ vs. ChAT+ after DA depletion: *P* < 0.0001; control ChAT- vs. ChAT- after DA depletion: *P* = 0.042; ChAT+ vs. ChAT- after DA depletion: *P* < 0.0001 (n-control ChAT+ = 58 neurons; n-control ChAT- = 146 neurons; n-DA-depleted ChAT+ = 65 neurons, n-DA-depleted ChAT- = 222 neurons; from 7 different animals in both control and after DA depletion; Kolmogorov-Smirnov test).

Calcium imaging data analyses are as follows: Every video recording was filtered offline to enhance contrast and was played to detect regions of interest (ROIs) by visual inspection using circular areas corresponding to somatic areas displaying GCaMP6f fluorescence fluctuations using a graphical user interface from ImPatch. We obtained a list of ROIs for each experiment. Time series of GCaMP6f or fluorescence signals were used as an indirect measure of neuronal activity, striatal firing neurons elicit calcium transients or Ca^2+^ events with fast increases of fluorescence, followed by slow decays. Simultaneous electrophysiological recordings show that action potentials occur during the fast rise (Pérez-Ortega et al., 2016; García-Vilchis et al., 2018; Lara-González et al., 2019; Duhne et al., 2021). Fluorescent transient amplitudes were normalized by the maximum ROI fluorescence. Calcium transients were defined as ΔF/F0, where ΔF was the increase in fluorescence (Fi–F0), and the background fluorescence, F0, is an area around each ROI. The positive portion of the time derivative, d(ΔF/F0)/dt indicates the fast rise of fluorescence and was taken as an indicator of neuronal firing for any given cell as long as it exceeded 2.5 standard deviations (SD) from the baseline signal (Pérez-Ortega et al., 2016). Inferred electrical activity was used to build activity matrices (raster plots) as binary rows with ones at the frames, where spikes were detected and zero otherwise (Figure 1 below). By stacking these rows of inferred spikes, we obtained a binary matrix whose dimensions are R × C, where R is the number of cells or active ROIs (y-axis or rows) and C is the number of frames (x-axis or columns/movie frames shown as time) of the experimental condition. The histogram at the right of the raster plots shows activity per cell along time (% frames active/total time). The coactivity histograms below the raster plots used column vectors with their summed cell activity on a frame-by-frame basis. The significance of coactivity peaks (dashed line) was obtained with Monte Carlo simulations (see Pérez-Ortega et al., 2016). A first metric is the rate of activity accumulation: the addition of these vectors along time (see Supplementary Figure 2). Linear fits were approximated to these additions, and their slopes were used in box plots to compare different experimental conditions (Aparicio-Juárez et al., 2019). Free-distribution Wilcoxon’s T rank-sum test was used for paired samples (before and after drug administration), Mann–Whitney U rank-sum test for unpaired samples (control vs. DA-depleted neurons), and Friedman or Kruskal–Wallis ANOVA for several samples to compare the rate of activity accumulation (see above). A second metric was the cumulative distribution function (CDF) using samples that included all neurons from all experiments under the same condition to see whether there is a difference with the smaller samples. CDFs probabilities were calculated using the percentage of active frames: the number of active frames divided by the total number of frames multiplied by 100. To compare CDFs we use Kolmogorov-Smirnov test, a non parametric statistic that does not require observations independence. In addition we used the Bonferroni method for multiple comparisons: obtaining the probability or less that a given neuron is active during the experimental condition. Displacements to the right denote increases in neuronal population activity.

## Results

### Cholinergic interneurons increase their excitability in dopamine-depleted conditions.

**FIGURE 2:**
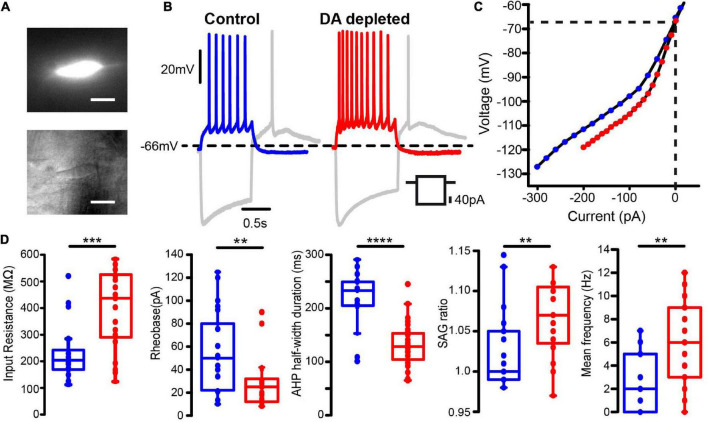
Increased excitability of dopamine depleted identified cholinergic interneurons revealed by intracellular current injections. **(A)** Top: Photomicrograph of a ChAT-positive neuron expressing GCaMP6f. Bottom: The same neuron viewed with infra-red (IR) microscopy (Scale bar: 10 μm) **(B)** left: Whole-cell patch clamp recordings of a representative cholinergic interneuron (CIN) from the control striatum. Right: Recordings of a CIN from dopamine (DA) depleted striatum (6-OHDA model). Voltage responses evoked by hyperpolarizing and depolarizing current injections are compared. **(C)** Representative current-voltage (I-V plot) relationships of CINs in each condition. **(D)** Box-plots comparing neuronal input resistance, rheobase, sag ratio and mean firing frequency upon 1 s, 40 pA depolarizing current for samples in both conditions. The half-width duration was measured during spontaneous firing at similar frequencies: 3–4 Hz (Mann–Whitney *U*-test: **P < 0.01; ***P = 0.0008;****P < 0.0001).

An example of an identified striatal CIN with GCaMP6f expression (Figure 2A, top) is the same neuron under infra-red microscopy (Figure 2A, bottom). It is shown that the identified neurons in this way exhibit electrophysiological properties previously reported for CINs in control conditions (Table 1): resting membrane potential around –60 mV (when they were found silent), a “sag” and rebound, broad action potentials (APs), followed by remarkable medium afterhyperpolarization (mAHP), and a slow afterhyperpolarization (sAHP) following a depolarizing current step (Jiang and North, 1991; Bennett and Wilson, 1999; Maurice et al., 2004; Wilson, 2005; Sanchez et al., 2011). Also, as previously reported, dopamine depletion enhances CINs firing response to similar depolarizing 1 s, 40 pA current steps (Figure 2B and Supplementary Figure 1), control: 2.6 ± 0.58 Hz, *n* = 21 cells from 9 different animals and DA-depleted: 5.7 ± 0.86 Hz, *n* = 21 cells from 11 different animals; *P* = 0.0098; Mann–Whitney *U*-test), and increases the “sag” ratio in response to a 1 s hyperpolarizing current step (-100 pA) in control: 1.019 ± 0.009; *n* = 33 cells from 14 different animals and DA-depleted: 1.053 ± 0.009; *n* = 32 cells from 15 different animals (Figure 2D; *P* = 0.0045; Mann–Whitney *U*-test). Current-voltage relationships (I–V plots) were built to measure CINs input resistance (R_*N*_) in both conditions (Figures 2C,D left) showing that R_*N*_ is significantly increased in DA-depleted neurons as compared to control conditions 235.6 ± 21.4 MΩ; control *n* = 28 cells from 11 different animals vs. 363.2 ± 28.0 MΩ; DA-depleted *n* = 29 cells from 13 different animals (*P* = 0.0008; Mann–Whitney *U*-test; see: Paz et al., 2021). The I–V plots confirm that DA depletion modifies CINs’ electrophysiological profile (Deng et al., 2007; Sanchez et al., 2011; Paz et al., 2021). The increase in R_*N*_ coincides with a lower rheobase: Control: 54.9 ± 7.8 pA, n = 21 cells from 9 different animals vs. DA-depleted: 30.4 ± 5.6 pA, *n* = 21 cells from 10 different animals (Figure 2D, 2nd frame; *P* = 0.0084; Mann–Whitney *U*-test; Fino et al., 2007), which explains, in part, the CINs hyperexcitability in the absence of dopamine modulation. Finally, we also found a significant decrease in AHP half-width duration in DA-depleted CINs as compared to controls (analyzed during spontaneous firing), control: 217 ± 14 ms, *n* = 16 cells from 7 different animals vs. DA-depleted: 130 ± 7 ms, *n* = 31 cells from 15 different animals; Figure 2D, 3rd frame; *P* < 0.0001; Mann–Whitney *U*-test), confirming that dopamine (DA) is an important modulator of CINs intrinsic properties, including the hyperpolarization-activated cation current or HCN as manifested by the “sag” during hyperpolarization (Aosaki et al., 1998; Maurice et al., 2004; Deng et al., 2007; Sanchez et al., 2011; Straub et al., 2014; Tubert et al., 2016; McKinley et al., 2019; Paz et al., 2021).

**TABLE 1:** Changes in intrinsic properties of CINs after DA-depletion.

Properties		Control	DA-depleted
*Action potential* Control (*n* = 16) *Indented (n* = *6)* *Smooth (n* = *10)* DA-depleted (*n* = 30) *Indented (n* = *5)* *Smooth (n* = *25)*	Threshold (mV) *Indented* *Smooth*	–41.23 ± 2.29 –40.52 ± 1.83	–39.88 ± 3.90 –41.09 ± 0.75
	Duration at half-width (ms) *Indented* *Smooth*	4.9 ± 0.76 5.2 ± 0.56	5.2 ± 0.81 4.1 ± 0.24
	Amplitude from threshold (mV) *Indented* *Smooth*	56.08 ± 1.87 47.22 ± 4.59	49.85 ± 5.16 55.07 ± 1.96
	Max. depolarization rate, dV/dt (mV/s) *Indented* *Smooth*	35.73 ± 4.26 41.69 ± 7.71	30.00 ± 4.23 54.38 ± 4.15
	Max. repolarization rate, dV/dt (mV/s) *Indented* *Smooth*	–12.37 ± 1.17 –11.73 ± 1.75	–10.08 ± 1.95 –14.39 ± 0.99
	mAHP amplitude (mV) *Indented* *Smooth*	7.18 ± 0.78 9.54 ± 1.38	4.61 ± 0.65* 8.89 ± 0.83
*mAHP (ms)* Control (*n* = 16) DA-depleted (*n* = 30)	Measured at half-width during spontaneous firing at similar frequencies (3–4 Hz)	216.9 ± 13.61	130.3 ± 7.02****
*Firing Frequency (Hz)* Control (*n* = 21) DA-depleted (*n* = 21)	Average number of APs in response to a 1 s, 40 pA, current injection	2.6 ± 0.58	5.71 ± 0.86**
*Input resistance* *(*MΩ*)* Control (*n* = 28) DA-depleted (*n* = 29)	Measured at –60 mV	235.6 ± 21.4	363.2 ± 28.02***
*Rheobase (pA)* Control (*n* = 21) DA-depleted (*n* = 21)	Minimal current to elicit one action potential	54.9 ± 7.8	30.4 ± 5.6**
*Sag ratio* Control (*n* = 33) DA-depleted (*n* = 32)	Vpeak at the beginning/Vpeak at the end of a –100 pA/1s step	1.019 ± 0.009	1.053 ± 0.009**
*Membrane potential* *(Vm)* Control (*n* = 28) DA-depleted (*n* = 12)	Measured during silent periods (mV)	–64.96 ± 1.01	–61.78 ± 1.77

Mann-Whitney U-test *P = 0.03; **P = 0.045; ***P = 0.0008; ****P < 0.0001.

**FIGURE 3:**
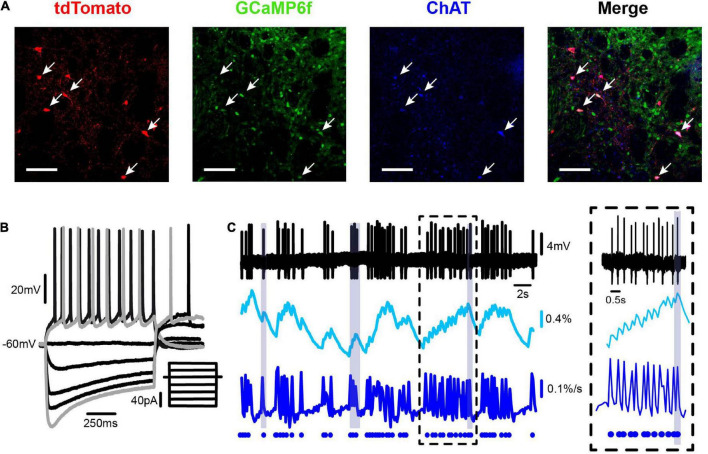
Simultaneous electrophysiological and calcium imaging recording of a striatal cholinergic interneuron. **(A)** Panels show from left to right: a double-photon micrograph of neurons expressing tdTomato (red), the same field showing neurons expressing GCaMP6f (green; under the synapsin promoter), neurons immunoreactive to anti-ChAT antibodies (blue), and the merge of previous panels showing that ChAT neurons correspond to the ones with tdTomato. **(B)** Whole-cell current clamp recording of a representative striatal CIN showing voltage responses to 1 s depolarizing and hyperpolarizing current injections at the soma: firing, inward rectification (“sag”), and rebound (inset illustrates stimulus currents from –80 to 30 pA in 10 pA steps). **(C)** Simultaneous on-cell extracellular recording of a spontaneous firing CIN (top), its corresponding calcium transients (ΔF/F0; 2nd trace), and inferred electrical activity from calcium fluorescence [d(ΔF/F0)/dt: third trace]. Dots at the bottom trace represent the way the inferred electrical activity is illustrated in the raster plots as in Figure 1. A neuron with bursts and pauses is shown to compare real (top) with inferred activity (bottom). Inset: zoom of recording at a time within the dashed square.

**FIGURE 4:**
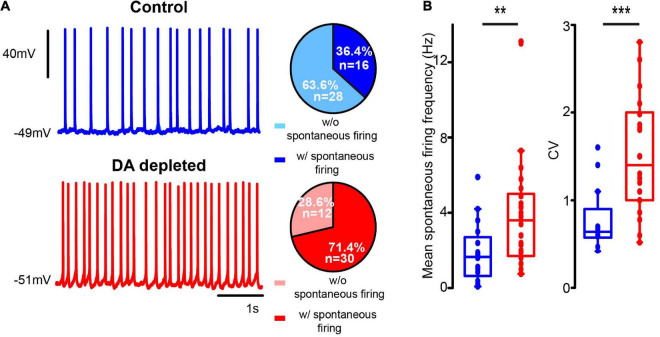
DA-depleted cholinergic interneurons had increased spontaneous firing. **(A)** Top: a representative whole cell patch clamp electrophysiological recording of a CIN exhibiting spontaneous firing (zero current) in control conditions. Bottom: a CIN exhibiting spontaneous firing (zero current) from the DA-depleted sample. Pie plots on the right show percentages of neurons found with and without spontaneous firing at the time of recording (****P < 0.0001; df = 1; χ^2^). **(B)** Box-plots show samples of mean firing rate obtained from 1 s of spontaneous firing from electrophysiological recordings (**P = 0.0061; Mann–Whitney U) and coefficient of variation (***P = 0.001; Mann–Whitney U) during spontaneous firing. Control *n* = 16 neurons from 7 different animals; DA-depleted *n* = 30 neurons from 14 different animals.

Commonly, CINs exhibiting spontaneous firing during on-cell patch (see Figure 3) kept exhibiting it after the whole-cell, either with perforated or unperforated patch modalities (Figure 4A). Enhanced excitability of the CINs population in DA-depleted conditions is also manifested by an increased percentage of neurons exhibiting spontaneous firing (*P* = 0.0001; *df* = 1; χ^2^ test). If found firing spontaneously, DA-depleted CINs had significant higher firing rates: in control: 1.88 ± 0.40 Hz; *n* = 16 cells from 7 different animals; DA-depleted: 3.94 ± 0.55; *n* = 30 cells from 14 different animals; *P* = 0.0061; Mann–Whitney *U*-test). The coefficient of variation also increased in DA-depleted neurons: control: 0.76 ± 0.08, *n* = 16 cells from 7 different animals; DA-depleted: 1.45 ± 0.11, *n* = 30 cells from 7 different animals (*P* = 0.001; Mann–Whitney *U*-test; Figure 4B). As seen above, some CINs with spontaneous firing may become silent and vice versa.

**FIGURE 5:**
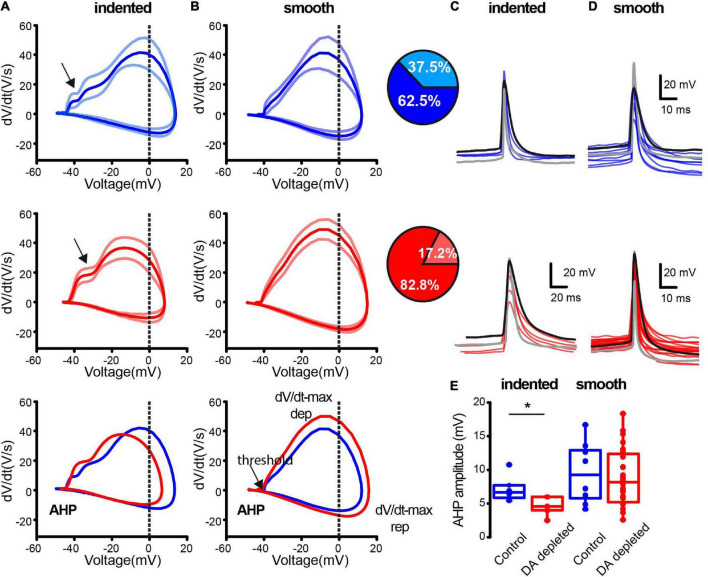
The extremes of a continuous spectrum of action potentials (APs) waveforms within the striatal population of cholinergic interneurons. **(A)** CINs with a clear indentation during AP depolarization as revealed by dV/dt(V)-plots (arrows). **(B)** CINs with a smoother depolarization during their AP. Top (blue) traces correspond to APs in control conditions and middle (red) traces correspond to APs from DA-depleted neurons. APs were chosen during spontaneous firing at a similar frequency and membrane potential was measured at the threshold. The dV/dt(V)-plots are illustrated as means ± standard error of the mean (SEM). Bottom traces in **(A,B)** superimpose means from control and DA-depleted neurons (Control-indented *n* = 6; Control-smooth *n* = 10 cells from 7 different animals; DA depleted- indented = 5; DA depleted –smooth *n* = 25 cells from 14 different animals). Insets: pie plots show the percentage of indented plots during AP depolarization in paler color; more neurons with indentation during depolarization of APs are present in control conditions (*P* = 0.0001; df = 1; χ^2^). **(C)** Stacked APs averaged from different neurons from indented AP depolarization in control (blue) and during DA depletion (red). **(D)** Superimposition of averaged APs without indentation. **(E)** Shown differences are non-significant except for AHP amplitude in indented samples (*P = 0.03; Mann–Whitney *U*-test).

We then asked whether differences in intrinsic properties were due to stimulation with intracellular currents that differentially activate distinct classes of ion channels (Deng et al., 2007; Tubert et al., 2016) or if they are also present when neurons are compared during spontaneous firing with no stimulus at similar membrane potentials and frequencies. Thus, AP features between control and DA-depleted CINs were analyzed with dV/dt(V)-plots (Bean, 2007). We found a spectrum of APs waveforms whose extremes are depicted in Figures 5A,B. Plots were averages ± SEM of several APs from single neurons with spontaneous firing chosen to be at the same frequency and similar membrane potentials as measured at firing during 2-min intervals (Control- indented *n* = 6; Control- smooth *n* = 10 cells from 7 different animals; DA depleted- indented *n* = 5; DA depleted-smooth *n* = 25 neurons from 14 different animals). Average traces from the control sample (top blue) have a clear indentation during depolarization of the AP, while other traces exhibit a smoother rise toward their peaks. Traces from the DA-depleted neurons sample also show indentation during depolarization while other neurons have a smoother rise (Table 1). The lighter color shows the percentage of APs with indentation during depolarization (comparison of percentages with and without indentation yield *P* = 0.0001; df = 1; χ^2^ test), suggesting that increases in firing rate are accompanied by fast-rising APs, supporting the view of changed intrinsic properties (e.g., ion channels redistribution) due to DA depletion. Indentation is commonly attributed to AP generation in the initial segment of the axon (Chand et al., 2015). Apparent differences shown in the superimposition of averaged traces from control and DA-depleted neurons were non-significant, since individual APs show great variability (Figures 5C,D). A significant difference was found in AHP amplitudes in CINs plots that showed the indentation (Figure 5E; *P* = 0.03; Mann–Whitney *U*-test). Several ion channels participate in the making of the AHP (Deng et al., 2007; Sanchez et al., 2011; Tubert et al., 2016).

### Activity of striatal cholinergic interneurons as observed with calcium imaging

To simultaneously follow the activity of dozens of CINs within striatal microcircuits of histological dimensions, we combined adenoviral infection of FLEX-tdTomato in ChAT-Cre mice to label CINs (ChAT-positive cells; Figure 3A, first frame from left to right) and have a reference photomicrograph, with Syn-GCaMP6f expressed in neurons of different classes inside the infection area (Figure 3A, second frame from left to right). The third frame in Figure 3A shows that tdTomato-labeled neurons were also immunoreactive to ChAT antibodies. The last image shows the merging of previous frames. Commonly reported electrophysiological profiles of CINs were identified in all tdTomato-positive cells recorded (Figure 3B). Figure 3C shows from top to bottom: extracellularly recorded APs from an identified CIN, the simultaneous recording of fluorescent signals from calcium imaging, the inferred electrical activity from fluorescent signals [d(ΔF/F0)/dt], and finally, the dots at the bottom show how the inferred activity is represented in the raster plots (Figure 1). Note the close similarity between real and inferred spikes. In this way, activity within a microcircuit can be illustrated using the raster plots (see section “Materials and Methods”), in which rows on the y-axis represent the activity of each neuron over time, while the x-axis represents movie frames expressed in time units.

Figure 1A shows a raster plot in control conditions. The dots in each row of the raster plot show the recorded activity of individual neurons belonging to different populations within the striatal microcircuit over time. Blue dots denote identified CINs (ChAT+), whereas gray dots show ChAT- neurons. The histogram at the right shows % activity cell by cell (rows: frames with activity/total frames times 100). The histogram at the bottom represents vertical summed activity column by column (frame by frame), which are the neurons that fire together (coactivity). The sum of this histogram along time denotes the rate of activity accumulation (see section “Materials and Methods” and Supplementary Figure 2), represented as the slopes of fitted straight lines (Figure 1C). This same arrangement is used in Figure 1B to represent the activity of the DA-depleted striatum. Red dots represent identified CINs, whereas black dots are ChAT- neurons. The threshold to consider a significant peak of coactivity after Monte Carlo simulations (Pérez-Ortega et al., 2016) is represented by a dotted line (*P* < 0.05). In control conditions, there are few or non-significant peaks of coactivity (Figure 1A, top; *n* = 7 slices from 7 different animals; Carrillo-Reid et al., 2008). In contrast, in DA-depleted conditions, neuronal activity is enhanced displaying significant peaks of spontaneous coactivity (Figure 1B; *n* = 7 slices from 7 different animals; Jáidar et al., 2010; Plata et al., 2013a; Aparicio-Juárez et al., 2019; Lara-González et al., 2019). In this condition, identified CINs underlie significant peaks of coactivity (red labeled in the coactivity histogram) regardless of representing a small percentage of striatal neurons. The rate of activity accumulation was significantly increased in ChAT+ neurons after DA depletion (Figure 1C; *P* = 0.0087; Kruskal-Wallis ANOVA with *post hoc* Dunn’s test). Cumulative distribution functions (CDFs) using all cells from the different samples show significant differences (Figure 1D): control ChAT+ vs. control ChAT- neurons: *P* = 0.76; control ChAT+ vs. ChAT+ after DA depletion: *P* < 0.0001; control ChAT- vs. ChAT- after DA depletion: *P* = 0.042; ChAT+ vs. ChAT- after DA depletion: *P* < 0.0001 (n-control ChAT+ = 58 neurons; n-control ChAT- = 146 neurons; n-DA depleted ChAT+ = 65 neurons, n-DA depleted ChAT- = 222 neurons; from 7 different animals in both control and after DA depletion; Kolmogorov-Smirnov test). To conclude, a multi-recording technique demonstrates that CINs increase their activity after DA depletion, and this increase is relatively larger than that of ChAT- neurons (Ding et al., 2006; Sanchez et al., 2011; Tubert and Murer, 2020; Paz et al., 2021). For this reason, the next figures show the raster plots of identified CINs.

**FIGURE 6:**
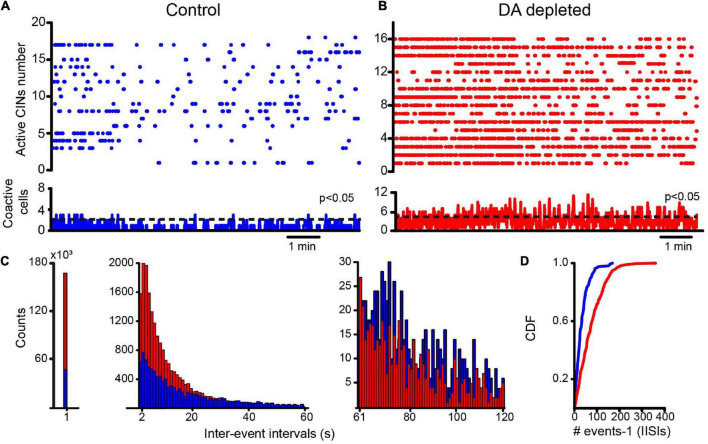
Striatal cholinergic interneurons increase their activity as shown by raster density and modify their firing pattern after DA depletion. **(A)** The raster plot showing the activity of several identified CINs in a brain slice in control conditions. A few significant coactivity peaks have 3 or fewer neurons. **(B)** The raster plot shows the activity of several identified CINs in DA-depleted conditions; augmented firing is clear during the simultaneous recording of CINs. Several significant coactivity peaks along time have 6 or more neurons (P = 0.05 after Monte Carlo simulations; see Pérez-Ortega et al., 2016). **(C)** The distribution of all inter-event intervals illustrates differences in raster plot density in both conditions (an event—a dot—denotes neural activity; an inter-event interval is silence between events). The distribution is skewed and has to be shown at different scales. The left histogram shows intervals of < 1 s showing that DA-depleted CINs activate more frequently. The middle histogram shows more prolonged events < 60 s. DA-depleted CINs have more events of this type since activity trains are followed or preceded by pauses. The right histogram shows that more prolonged intervals of < 120 s belong to CINs in control conditions. **(D)** Lumped and normalized as cumulative distribution functions (CDFs) of all inferred interspike intervals (IISIs), counting all intervals for each condition, show significant differences (*P* < 0.0001; control, *n* = 163 cells from *n* = 10 slices from 10 different animals; DA-depleted CINs, *n* = 285 cells from *n* = 13 slices from 12 different animals; Kolmogorov-Smirnov test). DA-depleted CINs appear to fire in trains with pauses exhibiting a higher density in the raster plots.

We used adenoviral infection of calcium indicator GCaMP6f under the ChAT promotor. This allowed us to exclusively follow CINs activity along time. Figure 6A shows a representative raster plot of CINs activity in control conditions: trains or single spikes are separated by silent periods of various durations. Figure 6B shows a representative raster plot of CINs activity in DA-depleted conditions. As in previous results (Figure 1), the appearance of numerous significant peaks of coactivity indicates CINs hyperactivity in DA-depleted conditions: more trains of spikes preceded or followed by pauses are seen, apparently increasing the density of firing in the raster plots. Figure 6C shows a histogram of all silent intervals regardless of the duration (density = 1/silent events of all durations, most being inferred interspike intervals). The histogram is skewed with a long tail and is illustrated divided into three different scales: at the left a histogram of all intervals less than 1 s shows that DA-depleted conditions have more inter-event intervals of this duration implying that CINs fire more frequently. In the middle, the histogram shows pauses between trains of activity. They appear more frequently in DA-depleted conditions, reflecting the numerous trains of spikes preceded or followed by pauses. The right histogram shows that more prolonged intervals between events belong to control conditions when neurons fire in tonic or irregular modes. To see whether these differences reflect a change in firing pattern, we normalized and lumped all inferred interspike intervals (IISIs) regardless of their duration in cumulative distribution functions (CDFs); clearly more pauses are found in DA-depleted conditions (*P* < 0.0001; control, *n* = 163 cells from *n* = 10 slices from 10 different animals; DA-depleted CINs, *n* = 285 cells from *n* = 13 slices from 12 different animals; Kolmogorov-Smirnov test), showing a change in firing pattern from tonic to more irregular or burst-like trains of spikes with pauses. Clearly, simultaneous recording of identified CINs shows the increased activity of most CINs during DA depletion, while individual neurons increase, decrease, or pause their firing. Therefore, reported discrepancies may be due to different sampling methods on single neurons (Ding et al., 2006; Sanchez et al., 2011; McKinley et al., 2019; Choi et al., 2020; Tubert and Murer, 2020; Paz et al., 2021), showing that population recordings yield a better illustration. Our next question was whether CINs firing during DA depletion also depends on extrinsic synaptic inputs known to be increased during Parkinsonism (Galarraga et al., 1987; Calabresi et al., 1996; Henderson et al., 2000; Zhang et al., 2013; Smith et al., 2014; Villalba et al., 2015; Glajch et al., 2016; Parker et al., 2016; Aceves-Buendia et al., 2017; Zhai et al., 2018).

### Synaptic inputs contribute to cholinergic interneurons hyperactivity in dopamine-depleted conditions

**FIGURE 7:**
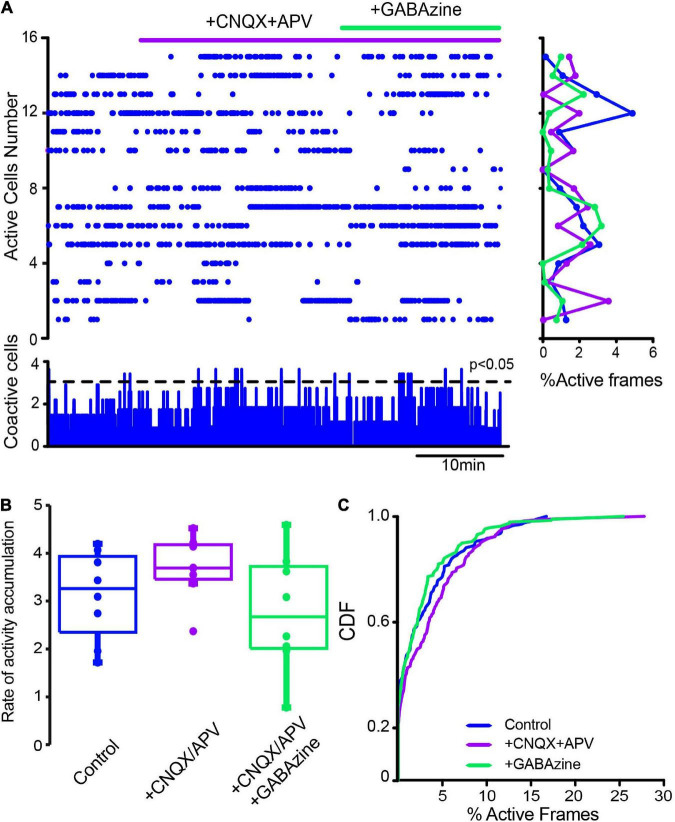
Influence of glutamatergic inputs on cholinergic interneurons’ spontaneous activity in control conditions without stimulation. **(A)** The raster plot shows the spontaneous activity of CINs in control conditions and after applying 10 μM CNQX and 50 μM APV. Thereafter 10 μM of GABAzine were administered. The histogram at the right shows activity per neuron (rows), histogram at the bottom shows activity per column (coactivity). **(B)** Box plots show samples of the rate of activity accumulation with non-significant changes (Wilcoxon’s T or Friedman tests). **(C)** Cumulative distribution functions (CDFs) of ChAT+ in control conditions and with the antagonists: CINs control vs. CINs + CNQX + APV: *P* = 0.087; + Gabazine: *P* = 0.24 (Kolmogorov-Smirnov test: n-CINS control = 116 cells, + CNQX + APV = 113; + Gabazine = 129 cells from *n* = 8 slices from 8 different animals).

**FIGURE 8:**
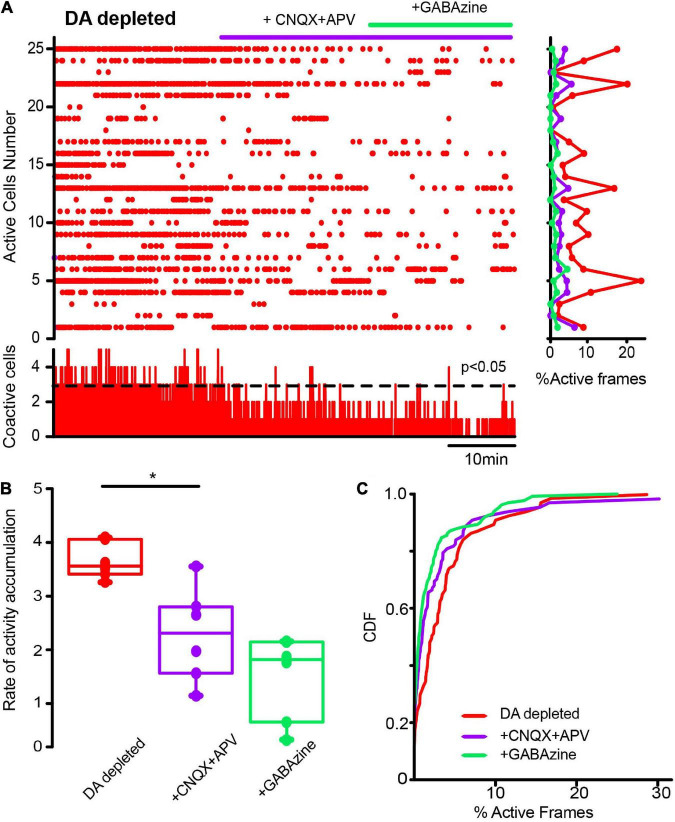
Glutamatergic inputs to the striatal microcircuit are essential for the activity increase in cholinergic interneurons in dopamine-depleted tissue. **(A)** The raster plot represents the activity of identified CINs recorded simultaneously in DA-depleted tissue, before and after applying glutamatergic antagonists (10 μM CNQX and 50 μM APV) and the GABAergic antagonist (10 μM GABAzine). Both cell activities (right: rows) and significant coactivity peaks (bottom) are decreased. **(B)** Box plots show significant reductions in the rate of activity accumulation after the antagonists (*P = 0.03; Wilcoxon *T*-test: DA-depleted vs. DA depleted with glutamatergic antagonists). Subsequent application of GABAzine appears to further reduce the rate of activity accumulation, but this is non-significant. **(C)** Cumulative distribution functions (CDFs) of ChAT+ neurons: DA-depleted CINs vs. CINs plus CNQX + APV (*P* < 0.0001); with further addition of GABAzine (*P* < 0.0001; Kolmogorov-Smirnov test; n-DA-depleted CINs = 159 neurons; with CNQX + APV = 145 neurons; plus GABAzine = 143 neurons from *n* = 6 slices from 6 different animals).

We investigated whether changes in striatal synaptic inputs could also contribute to pathological CINs hyperactivity. To this end, glutamatergic and GABAergic antagonists (10 μM CNQX + 50 μM APV and 10 μM Gabazine) were administered in different orders to observe the contribution of extrinsic inputs to CINs activity before and after DA depletion. Figure 7A shows a raster plot with CINs’ spontaneous activity in control conditions exhibiting scarce significant peaks of CINs coactivity (histogram at the bottom) or individual neuron activity (histogram at right). Note that activity in the control is scarce, and administration of glutamatergic antagonists (+ CNQX + APV) did not change it significantly. In fact, neither the rate of activity accumulation (Figure 7B; *P* > 0.1; Wilcoxon *T*-test) nor the cumulative distribution functions (CDFs; Figure 7C; *P* > 0.05; Kolmogorov-Smirnov test; n-Control CINs = 116 neurons; CNQX + APV = 113 neurons; plus Gabazine = 129 neurons; from *n* = 8 slices from 8 different animals) show significant changes, suggesting that excitatory glutamatergic inputs from cortex and thalamus are at its minimum when the control striatum is at rest or not activated (Lara-González et al., 2019), in opposition to control-activated striatum (Pérez-Ortega et al., 2016). In contrast, CINs appear hyperactive after DA depletion (Figure 8), showing various significant peaks of CINs’ coactivity (histogram at the bottom). The addition of glutamatergic antagonists reduced this hyperactivity, peaks of coactivity, and individual cell activity (histogram at right). In fact, the rate of activity accumulation is significantly decreased (Figure 8B; *P* = 0.03; Wilcoxon *T-*test). Subsequent addition of the GABAergic antagonist, Gabazine, has a non-significant tendency to further reduce CINs hyperactivity in DA-depleted conditions. Pooling neurons from all experiments at different conditions to build cumulative distribution functions (CDFs) also show significance: DA-depleted CINs vs. DA-depleted CINs plus CNQX + APV: *P* < 0.0001; with further addition of Gabazine (Figure 7C; *P* < 0.0001; Kolmogorov-Smirnov test; n-DA-depleted CINs = 159 neurons; DA-depleted CINs with CNQX + APV = 145 neurons; plus Gabazine = 143 neurons; from *n* = 6 slices from 6 different animals). We conclude that a great part of CINs hyperactivity found after DA depletion is due to glutamatergic transmission either from the cortex or thalamus (e.g., Arias-García et al., 2017). Because in control non-stimulated conditions glutamatergic blockade has non-significant actions, it is inferred that a change in synaptic glutamatergic transmission due to DA depletion affects CINs activity (Villalba et al., 2015, 2019; Parker et al., 2016; Aceves-Buendia et al., 2017).

**FIGURE 9:**
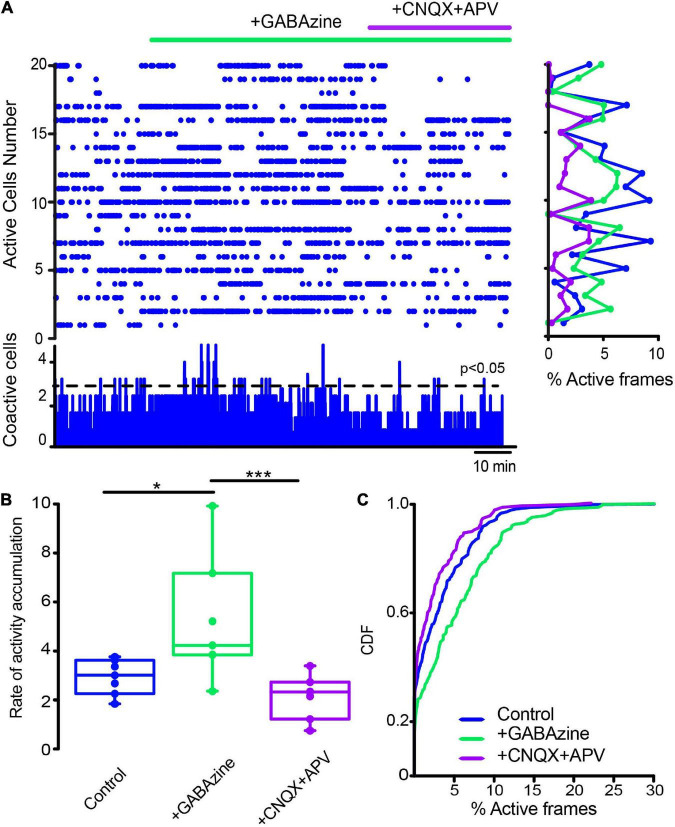
Global actions of inhibitory GABAergic inputs during CINs spontaneous activity in control conditions. **(A)** Raster plot represents the simultaneous activity of CINs before and after applying 10 μM GABAzine: an increase in activity is reflected cell by cell (rows activity at right histogram) and is significant at the level of coactivity peaks (histogram at the bottom; *P = 0.039; Wilcoxon T). An addition of 10 μM CNQX + 50 μM APV appears to decrease this activity. **(B)** Box plots show the rate of activity accumulation. Blockade of glutamatergic transmission does inhibit activity after GABAzine (***P = 0.0002; Friedman ANOVA with *post hoc* Dunn’s test). **(C)** Cumulative distribution functions (CDFs) of activity of ChAT+ identified neurons in control conditions and during synaptic antagonists: control vs. + GABAzine: *P* = 0.0033; control vs. + GABAzine + CNQX + APV: *P* = 0.052; and Gabazine vs. + Gabazine + CNQX + APV: *P* < 0.0001. (n-control = 111 neurons; + Gabazine = 133 cells; + Gabazine + CNQX + APV = 116 cells from *n* = 7 slices from 7 different animals; Kolmogorov Smirnov test).

**FIGURE 10:**
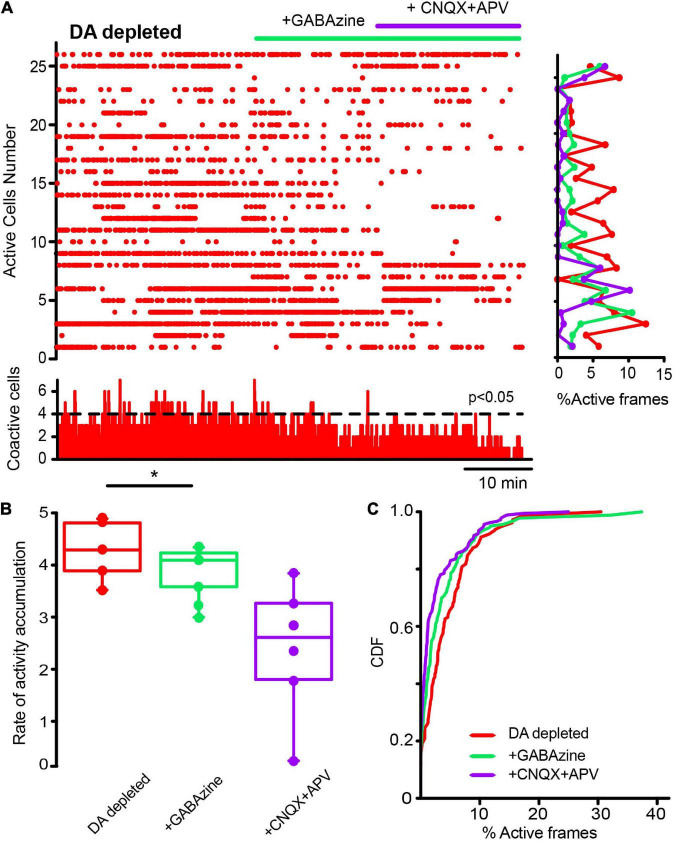
Inhibitory GABAergic inputs also contribute to cholinergic interneurons hyperactivity in dopamine-depleted tissue. **(A)** The raster plot represents the activity of identified CINs recorded simultaneously in a DA-depleted striatal slice. Applying 10 μM of GABAzine decreased hyperactivity contrary to control tissue (cf. Figure 9), where inhibition blockade increases neuronal activity as expected. In DA-depleted tissue, activity decreases cell by cell (rows in the histogram at right) and in significant peaks of coactivity (histogram at the bottom). An addition of 10 μM CNQX and 50 μM APV appears to further decrease this activity as excitation blockade is expected to do. **(B)** Box plots show that the rate of activity accumulation is significantly decreased by GABAzine (*P = 0.03; Wilcoxon T). **(C)** CDFs before and after addition of synaptic antagonists on CINs hyperactivity after DA depletion: DA-depleted CINs vs. CINs with addition of GABAzine: *P* = 0.0013; plus, addition of CNQX + APV: *P* < 0.0001; CINs + GABAzine vs. CINs with further addition of CNQX + APV: *P* = 0.0029 (n-DA depleted-CINs = 163 neurons; + GABAzine = 155 neurons; + CNQX + APV = 149 neurons from *n* = 6 slices from 6 different animals; Kolmogorov-Smirnov test).

Next, we first administered a blocker of GABAergic inhibitory striatal synapses, 10 μM Gabazine, to find out if inhibition contributes to the scarcity of neuronal activity in the control non-stimulated striatum. Figure 9A shows, as expected, increases in control neuronal activity reflected in an increase in the rate of activity accumulation (Figure 9B; *P* = 0.039; Wilcoxon T), showing that inhibition contributes to low striatal activity. Further, if in this condition, glutamatergic antagonists were administered (10 μM CNQX + 50 μM APV), low spontaneous activity is restored (Figure 9B; *P* = 0.0002; Friedman ANOVA with *post hoc* Dunn’s test), suggesting that GABAergic synapses are controlling the spontaneous excitatory entries; thus, achieving excitatory-inhibitory balance for this nucleus. CDFs confirmed this view: after Gabazine inhibition activity is enhanced and restored after excitatory blockade (Figure 9C; control vs. + GABAzine; *P* = 0.0033; control vs. + GABAzine + CNQX + APV; *P* = 0.052; and + Gabazine vs. + Gabazine + CNQX + APV; *P* < 0.0001. (n-control = 111 neurons; + Gabazine = 133 cells; + Gabazine + CNQX + APV = 116 cells from *n* = 7 slices from 7 different animals; Kolmogorov Smirnov test). Surprisingly, Figure 10 shows that CINs hyperactivity during DA-depleted conditions is decreased after Gabazine (Figure 10B; *P* = 0.03; Wilcoxon *T*-test). CDFs show the result of pooling all neurons from different samples: DA-depleted CINs vs. CINs with addition of Gabazine: *P* = 0.0013; and addition of CNQX + APV; *P* < 0.0001; CINs + Gabazine vs. CINs, with further addition of CNQX + APV: *P* = 0.0029 (n-DA depleted CINs = 163 neurons; + Gabazine = 155 neurons; + CNQX + APV = 149 neurons from *n* = 6 slices from 6 different animals; Kolmogorov-Smirnov test). We conclude that, paradoxically, the inhibitory transmission also contributes to CINs hyperactivity in the DA-depleted circuit; this result needs working hypotheses (see section “Discussion”) in view that control-tissue inhibitory blockade increases CINs activity as expected. On the other hand, blockade of excitatory glutamatergic transmission decreases CINs activity as expected. These results suggest that GABAergic transmission is also disrupted due to DA depletion (Nitsch and Riesenberg, 1995; Sullivan et al., 2008; Dehorter et al., 2009; Gonzales et al., 2013; Sato et al., 2014; Glajch et al., 2016; Dorst et al., 2020; Dong et al., 2021), provoking an excitatory-inhibitory imbalance that perhaps explains hyperactivity during DA depletion.

**FIGURE 11:**
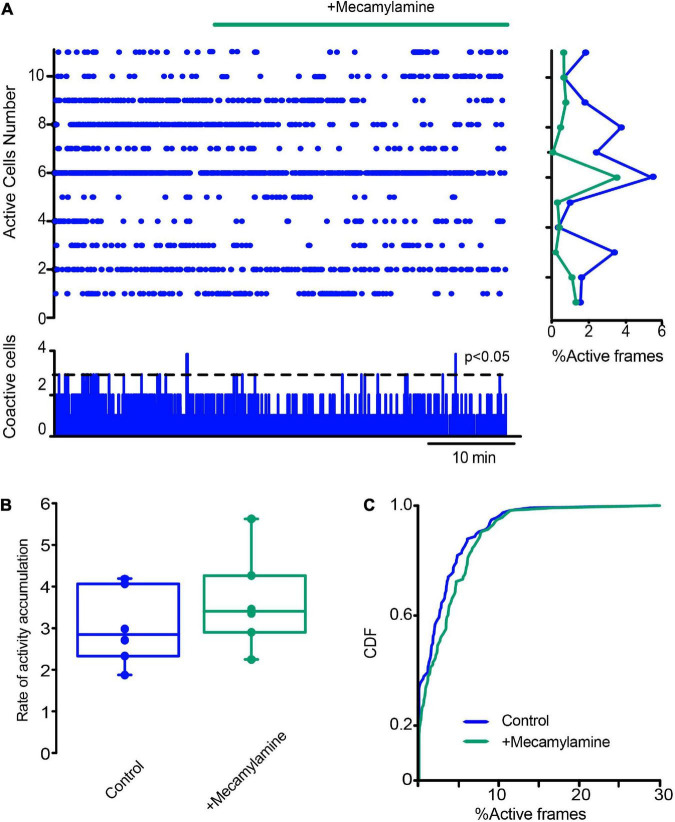
Blockade of nicotinic receptors does not have an obvious influence on CINs activity in control conditions. **(A)** The raster plot shows the simultaneous activity of CINs before and after applying the nicotinic receptor antagonist mecamylamine (10 μM). **(B)** Box plots of the rate of activity accumulation. **(C)** Cumulative distribution function of activity of ChAT+ neurons with and without mecamylamine: *P* = 0.063 (Kolmogorov-Smirnov test: CINs control = 77 neurons; with mecamylamine = 97 cells; from *n* = 6 slices from 6 different animals).

**FIGURE 12:**
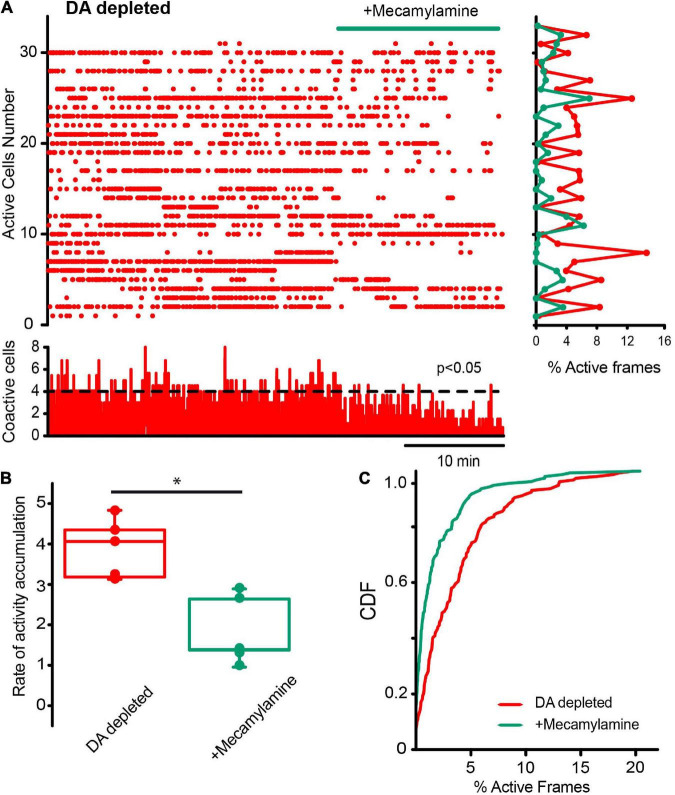
Cholinergic synaptic transmission *via* nicotinic receptors is also important to maintain cholinergic interneurons hyperactivity in DA-depleted tissue. **(A)** Raster plot shows CINs hyperactivity in DA-depleted conditions. The addition of 10 μM of mecamylamine, a nicotinic receptor antagonist, appears to decrease this hyperactivity neuron by neuron (histogram at right) and in significant peaks of coactivity (histogram at the bottom). **(B)** Box plots show that the rate of activity accumulation along time is, in fact, decreased (*n* = 6; *P* = 0.05; Wilcoxon *T*-test). **(C)** CDFs show differences between activity of CINs during DA-depleted conditions before and after mecamylamine: *P* < 0.0001 (n-DA depleted CINs = 148 neurons; n-plus mecamylamine = 141 neurons, from *n* = 6 slices from 6 different animals: Kolmogorov-Smirnov test).

Finally, we decided to see another fast ligand-gated synapses present in the striatum: the cholinergic nicotinic synapses, not present in SPNs but expressed in CINs, other interneuron types, cortico-striatal, and thalamo-striatal terminals, since a polysynaptic inhibitory network among CINs, through nicotinic receptors, has been previously described to be modulated by DA D2-class receptors (Sandor et al., 1991; Sullivan et al., 2008; Abudukeyoumu et al., 2018; Dorst et al., 2020). The raster plot in Figure 11A shows CINs activity in control tissue before and after the addition of 10 μM mecamylamine, a non-specific nicotinic receptor antagonist, into the saline bath: activity is unchanged, suggesting that these synapses do not play a population role in control non-stimulated striatum. CDFs show a *p*-value = 0.063 (Kolmogorov-Smirnov test: CINs control = 77 neurons; with mecamylamine = 97 cells; from *n* = 6 slices from 6 different animals). This is opposed to what happens during DA depletion: Figure 12 shows that mecamylamine decreases individual cell activity, as well as co activity after DA depletion. The rate of activity accumulation and CDFs of DA-depleted CINs show a significant reduction after nicotinic receptors blockade (Figure 12B; *P* = 0.05; Wilcoxon *T*-test; Figure 12C; *P* < 0.0001; Kolmogorov-Smirnov test; n-DA depleted CINs = 163 neurons; n-DA-depleted CINs plus mecamylamine = 141 neurons, from *n* = 6 slices from 6 different animals). In conclusion, cholinergic nicotinic synapses also contribute to CINs hyperactivity after DA depletion, and it is an open question whether this transmission is also altered during Parkinsonism (Kaiser and Wonnacott, 2000; Azam et al., 2003; Bohr et al., 2005; Quik et al., 2009; Luo et al., 2013; Abudukeyoumu et al., 2018; Licheri et al., 2018).

Taken together, the present results show that fast ligand-gated synapses of the striatal circuitry contribute to CINs hyperactivity after DA depletion. Future research will find specific sources of these synapses as well as direct and indirect actions on CINs activity.

## Discussion

The altered activity exhibited by CINs during Parkinsonism is relevant due to its relation with the hypercholinergy that accompanies DA depletion (Barbeau, 1962; Aosaki et al., 1994, 1998; Yan et al., 1997; Galarraga et al., 1999; Pisani et al., 2000; Tanimura et al., 2017, 2019). The cholinergic-dopaminergic balance appears to be essential for normal striatal circuitry (Aosaki et al., 2010); its alteration is reflected in abnormal oscillatory activity and motor dysfunction (Magill et al., 2001; Courtemanche et al., 2003; Brown, 2007; Hammond et al., 2007; Moran et al., 2011; Lemaire et al., 2012; Cai et al., 2021; Iskhakova et al., 2021). Direct inhibition of CINs altered activity in PD-animal models shows restoration of motor behaviors reducing akinesia and bradykinesia (Maurice et al., 2015), while their opto-activation increases beta activity in striatal microcircuit; a PD hallmark (Kondabolu et al., 2016). These observations point to the upregulation of CINs activity under DA-depleted conditions, although discrepancies remain in this crucial point (Ding et al., 2006; Sanchez et al., 2011; Tubert et al., 2016; McKinley et al., 2019; Choi et al., 2020; Tubert and Murer, 2020; Paz et al., 2021). Differences with the present study may be explained because single-cell recordings are done in a period, in which the neuron could be firing or not. With simultaneous recordings, we observed that CINs have alternating periods of firing and silence. Moreover, some neurons go to silence while others enter firing. However, after minutes of recording, the overall population activity is globally increased after dopamine depletion. Also, we recorded spontaneous and not induced firing. The distribution of pauses between firing is significantly greater in DA-depleted conditions, mainly due to pauses between spike trains, although total firing is decidedly increased for longer periods compared to controls, due to more frequent spike trains and single spikes, hence, promoting an increase in the density of the raster plots (see section “Results”; Figure 6).

As previously reported, intrinsic properties of CINs contribute to their increase in activity and hypercholinergy (Galarraga et al., 1999): an increased input resistance and decreased rheobase in the absence of dopamine modulation (Fino et al., 2007) induce increased excitability. Intensity-frequency plots show that these differences influence CINs output to similar stimuli, and despite different constraints to avoid stimulation at arbitrary holding potentials, a reduction in the half-width duration of the AHP is seen in CINs after DA depletion, as measured during spontaneous firing at similar membrane potentials and frequencies in control and DA-depleted conditions (Wilson, 2005; Deng et al., 2007; Sanchez et al., 2011; Tubert et al., 2016; Paz et al., 2021). Finally, indentation during AP depolarization, suggesting the generation of action potentials in the initial segment, was reduced after DA depletion (Bennett and Wilson, 1998). All these observations support the hypotheses that changed intrinsic properties contribute to CINs hyperactivity during DA depletion.

However, the effect of fast ligand-gated synaptic entries onto CINs is just in the beginning stage of being studied. The main purpose of this work is to call attention to this issue and the challenges it carries for future investigation. Here, we explain and discuss our results proposing working hypotheses, perhaps, useful for future research. In this respect, the original findings of this work are detailed below.

### Actions of glutamatergic afferents on dopamine depleted cholinergic interneurons hyperactivity

Blockade of glutamatergic transmission significantly decreases CINs’ pathological hyperactivity; when in control conditions, it does not appear to alter CINs’ spontaneous activity (cf. Figures 7, 8). Glutamatergic sources mainly involve both cortex and thalamus (Lapper and Bolam, 1992; Consolo et al., 1996; Bernard et al., 1997; Bolam et al., 2000; Matsumoto et al., 2001; Minamimoto and Kimura, 2002; Ellender et al., 2013; Smith et al., 2014; Kosillo et al., 2016; Assous and Tepper, 2019) of which, thalamic input is the strongest in control conditions (Sidibé and Smith, 1999; Ding et al., 2010; Guo et al., 2015). Increased glutamatergic activity has been previously reported during the Parkinsonian state (Galarraga et al., 1987; Calabresi et al., 1996; Henderson et al., 2000; Zhang et al., 2013; Smith et al., 2014; Zhai et al., 2018), perhaps, due to a plasticity dysfunction of glutamatergic striatal entries (Villalba et al., 2015, 2019; Parker et al., 2016; Aceves-Buendia et al., 2017). Numerous posited anti-Parkinsonian drugs, such as amantadine and ketamine, aim to reduce these entries to the striatum (Allers et al., 2005; Bartlett et al., 2020). Here, we demonstrate that a suspected possible target for these entries is indeed the CINs (Lapper and Bolam, 1992; Calabresi et al., 1996; Consolo et al., 1996; Bernard et al., 1997; Sidibé and Smith, 1999; Matsumoto et al., 2001; Williams et al., 2002; Costa et al., 2006; Parr-Brownlie et al., 2009; Ellender et al., 2013; Doig et al., 2014), leaving the question of what source of these entries is more important in the pathological situation: thalamic or cortical (Arias-García et al., 2017), and whether the CINs are the main target as compared to SPNs or additional interneuron types (see below).

### Actions of GABAergic inputs on dopamine-depleted cholinergic interneurons hyperactivity

Surprisingly, blockade of GABAergic inputs also significantly decreases pathological CINs hyperactivity. When in the control condition, GABAergic blockade does what it is supposed to do: an increase in activity due to the unbalance between excitatory and inhibitory inputs (cf. Figures 9, 10). How to advance a working hypothesis to explain this result? One alternative is disinhibition, while the other possibility is that some GABAergic interneuron becomes excitatory. Weights of the different GABAergic inputs need not be the same and Parkinsonism could have distorted them, as the comparison with control conditions appear to suggest. Thus, an increase in glutamatergic transmission may lead to the hyperactivation of CINs, and in a feed-forward way, activation of CINs could promote the activation of presynaptic glutamatergic terminals (cortical and thalamic) through nicotinic receptors (Abudukeyoumu et al., 2018), as well as GABAergic inputs that have special features: LTS interneurons may further excite CINs through nitric oxide generating strong depolarizations (Elghaba et al., 2016), and in contrast, some NPY+ and TH+ interneurons may induce a strong inhibition through volume transmission (Tepper et al., 2018; Dorst et al., 2020). Therefore, not all GABAergic inputs appear to have the same weight or strong hyperpolarizations (coming from GABAergic interneurons or GABAergic neurons from the GPe; Raz et al., 2001; Mallet et al., 2012, 2016; Abdi et al., 2015; Fujiyama et al., 2015; Glajch et al., 2016; Aristieta et al., 2021; Cui et al., 2021), accompanied with strong depolarizations, would promote rebound in CINs activity and sequences of fire and pauses, even causing them to burst, generating hyperactivity. The GABAergic blockade could stop this type of firing decreasing overall activity. This is a hypothetical, but testable scenario.

### Actions of cholinergic nicotinic inputs on dopamine-depleted cholinergic interneurons hyperactivity

Blockade of cholinergic transmission *via* nicotinic receptors (nAChRs) was also able to reduce CINs hyperactivity (cf. Figures 11, 12). The actions of nAChRs in the striatal microcircuit are extensive. Different types of nAChRs are expressed presynaptically in dopaminergic and glutamatergic afferents that come from the substantia nigra compacta, thalamus, and cortex, respectively (Hill et al., 1993; Kaiser and Wonnacott, 2000; Zhou et al., 2001; Parker et al., 2004; Rice and Cragg, 2004; Zhang and Sulzer, 2004; Bohr et al., 2005; Campos et al., 2010; Licheri et al., 2018; Tanimura et al., 2019; Assous, 2021). In the cortico-striatal terminals, they promote glutamate release and transmission. Therefore, the most parsimonious working hypothesis to explain nicotinic receptor antagonist actions is that they reduce glutamate release from hyperactive afferents. This needs demonstration by future research.

Nonetheless, other mechanisms may also play a role. The nAChRs are expressed by CINs themselves (Azam et al., 2003; Abbondanza et al., 2022), and given their absence in SPNs, a network of CINs recruited through nAChRs may bring strong support to the highly recurrent pathological state found during DA depletion (Jáidar et al., 2010; Pérez-Ortega et al., 2016). This hypothetical CINs network also needs to be demonstrated. However, Figure 5 shows an increase in the coupling of CINs activity with the Parkinsonian hyperactivity observed in the microcircuit in general (Jáidar et al., 2010; Plata et al., 2013a), and this coupling appears to be absent in control conditions.

Finally, striatal GABAergic interneurons also express nAChRs (Koós and Tepper, 2002; Luo et al., 2013; Plata et al., 2013b; Ibáñez-Sandoval et al., 2015; Faust et al., 2016; Assous, 2021; Abbondanza et al., 2022) and produce SPNs inhibition *via* CINs. Thus, inhibition of any known interneuron class may be responsible for Parkinsonian hyperactivity in the striatal circuitry. Nevertheless, a special combination of CINs with LTS interneurons deserves attention, because synchronized trains of activity of LTSIs, may accompany the trains of activity generated by CINs (Dehorter et al., 2009; Elghaba et al., 2016). Nicotinic receptor activation of LTSIs by CINs is reciprocated by nitric oxide activation of CINs by LTSIs (Elghaba et al., 2016). Because cholinergic nicotinic activation may involve other GABAergic afferents and sources, this relationship may generate the trains of spikes interspersed by pauses seen in the Parkinsonian condition (Figure 6). In addition, Figure 5 shows that, although an increase in ChAT- neurons rate of activity accumulation is not-significant, the variance in the activity of these neurons is augmented, and CDFs between them were significantly different, suggesting that other neuron classes within the ChAT- population are also participating preferentially in microcircuit hyperactivity during the Parkinsonian state. Accordingly, stimulation of nicotinic receptors has been reported to reduce pathological activity and motor signs (Quik et al., 2009; Plata et al., 2013b), while their blockade decreases CINs hyperactivity.

## Conclusion

To summarize, the results of the present experimental work open several questions for future research, given the actions of fast ligand-gated synapses onto CINs Parkinsonian hyperactivity, and only future investigations will decide whether any of these hypothetical mechanisms is the main cause or a contributor to the generation of the highly recurrent ensemble that causes striatal hyperactivity during Parkinsonism. However, these results also point toward striatal CINs as potential main targets for therapeutic procedures.

## Data availability statement

The raw data supporting the conclusions of this article will be made available by the authors, without undue reservation.

## Ethics statement

The animal study was reviewed and approved by the Institutional Committee for Laboratory Animals Care and Use of the Instituto de Fisiología Celular (IFC), UNAM (NOM-062-Z00-1999; laboratory protocols JBD-59-15).

## Author contributions

MP-O and MD: study conception and design, acquisition of data, analysis and interpretation of data, and drafting of the manuscript. AF-S: acquisition of data and drafting of the manuscript. AO: data analysis. EG, JB, and EL-G: study conception and design, data interpretation, and critical revision. All authors contributed to the article and approved the submitted version.

## Footnotes

1
https://www.jax.org/

2
http://n2t.net/addgene:100837

3
http://n2t.net/addgene:100833

4
http://n2t.net/addgene:51503

5
http://impatch.ifc.unam.mx/

6
https://www.merckmillipore.com/

7
https://www.jacksonimmuno.com/

8
http://www.mathworks.com/products/matlab/
