# Supplementary material for: Synaptic determinants of cholinergic interneurons hyperactivity during parkinsonism
Source: Front Synaptic Neurosci. 2022 Sep 6;14:945816. doi: 10.3389/fnsyn.2022.945816 (PMC9485566; doi:10.3389/fnsyn.2022.945816)
Supplement: Supplementary file 1 [file Data_Sheet_1.docx]

**Supplementary data. Padilla-Orozco et al**


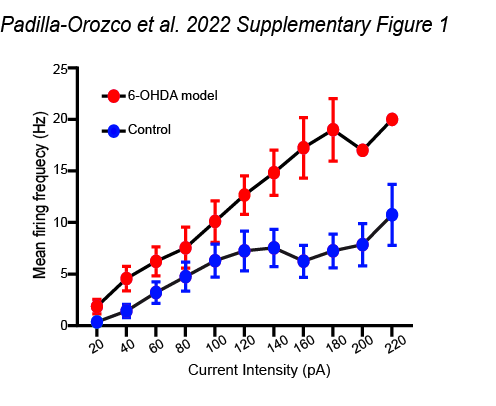


**Supplementary figure 1.** Intensity-frequency plots of cholinergic interneurons in control and after DA depletion (6-OHDA model of parkinsonism). Mean firing frequency to the same 1s stimuli are significantly increased (n=14 neurons from different animals for each sample; P<0.0001; two-way ANOVA with post hoc Tukey tests for multiple comparisons).


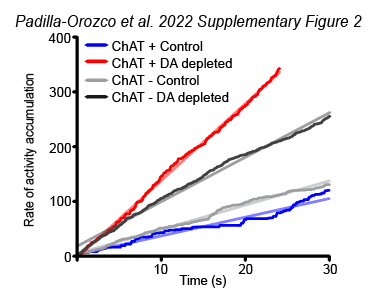


**Supplementary figure 2.** Representative traces showing that cholinergic interneurons exhibit a higher rate of activity accumulation after DA depletion. Rate of accumulation box plot graphs from single experiments were built by adding column vectors activity along time from the histograms of coactivity (at the bottom of raster plots). The slopes of fitted straight lines are plotted in box plots as rate of activity accumulation (e.g.: Fig. 5).
